# Dental anomalies in individuals with osteogenesis imperfecta: a systematic review and meta-analysis of prevalence and comparative studies

**DOI:** 10.1590/1678-7757-2023-0040

**Published:** 2023-09-04

**Authors:** Heloisa Vieira PRADO, Enio Cássio Barreto SOARES, Natália Cristina Ruy CARNEIRO, Ivanete Cláudia de Oliveira VILAR, Lucas Guimarães ABREU, Ana Cristina BORGES-OLIVEIRA

**Affiliations:** 1 Universidade Federal de Minas gerais Faculdade de Odontologia Departamento de Odontologia Social e Preventiva Belo Horizonte Minas Gerais Brasil Universidade Federal de Minas gerais, Faculdade de Odontologia, Departamento de Odontologia Social e Preventiva, Belo Horizonte, Minas Gerais, Brasil.; 2 Universidade Federal de Minas gerais Faculdade de Odontologia Departamento de Odontopediatria Belo Horizonte Minas Gerais Brasil Universidade Federal de Minas gerais, Faculdade de Odontologia, Departamento de Odontopediatria, Belo Horizonte, Minas Gerais, Brasil.

**Keywords:** Dental anomalies, Oral health, Osteogenesis imperfecta, Rare diseases

## Abstract

**Background:**

Osteogenesis imperfecta (OI) is a rare genetic disorder primarily caused by mutations in the genes involved in the production of type 1 collagen. OI is also known as brittle bone disease.

**Objective:**

This study aims to describe the prevalence of dental anomalies (except dentinogenesis imperfecta) in individuals with OI, and compare the prevalence of dental anomalies between individuals with and without OI and between individuals with different types of OI.

**Search methods:**

Searches in PubMed, Web of Science, Scopus, Ovid, and gray literature were performed in October 2022.

**Selection criteria:**

Observational studies (with or without a comparison group) that evaluated the prevalence of dental anomalies in individuals with OI. Data collection and analysis: Data items were extracted by two authors. Quality assessment employing the Joanna Briggs Institute checklists and meta-analyses was conducted. Results were provided in prevalence values and odds ratio (OR) / 95% confidence interval (CI). Strength of evidence was determined.

**Results:**

Eighteen studies were included. Most prevalent dental anomalies in individuals with OI included pulp obliteration (46.4%), dental impaction (33.5%), dental impaction of second molars (27%), and tooth agenesis (23.9%). Individuals with OI type III/IV had 20.16-fold greater chance of exhibiting tooth discoloration in comparison with individuals with OI type I (CI: 1.10–370.98). In comparison with the group without OI, the individuals with OI had 6.90-fold greater chance of exhibiting dental impaction (CI: 1.54–31.00). High methodological quality was found in 47% of the studies. Strength of evidence was low or very low.

**Conclusions:**

Pulp obliteration, dental impaction, and tooth agenesis were the most prevalent dental anomalies in the OI group. Individuals with OI were more likely to have dental impaction than individuals without OI. Individuals with OI type III/IV (severe-moderate) are more likely to have tooth discoloration than individuals with OI type I (mild).

## Introduction

Osteogenesis imperfecta (OI) is a rare genetic disorder with skeletal involvement. The estimated incidence of OI is one in every 15,000 to 20,000 live births.^1 , 2^ The OI corresponds to a heterogeneous group of hereditary diseases, the majority of which are autosomal dominant conditions with mutations in one of the *COL1A1* and *COL1A2* genes.^1 , 3^ These genes encode α1(I) and α2(I) chains of type I collagen, which is a fibril-forming collagen found in most connective tissues and abundant in bone, dentin, cornea, dermis, and tendon.^4^ Mutations can form low collagen (quantitative mutations) or structurally defective collagen (qualitative mutation), responsible for a more severe skeletal phenotype.^1 - 3^

Recently, rare autosomal recessive or X-linked mutations have been identified. In these cases, the genes are involved in extracellular post-modification of collagen (e.g. *CRTAP, LEPRE1* and *PPIB* ), collagen folding and intracellular trafficking (e.g. *SERPINH1* and *FKBP10* ), ossification or mineralization (e.g. *SERPINF1* ), and osteoblast development (e.g. *WNT1, CREB3L1* and *SP7* ).^1 , 2 , 5^

Generally, defects in type 1 collagen secretion result in insufficient osteoid production, affecting both endochondral and intramembranous ossification.^5^ Thus, the main manifestation of OI is bone frailty, which causes delayed growth and fractures throughout life. Joint laxity, bluish sclera, and hearing loss are common findings in affected individuals.^1 - 3 , 5^ Some individuals may also be affected by valve insufficiencies and aneurysms.^3^ Patients with OI have no mental deficits and they must be treated according to their age and not to their height.^3^

Due to its clinical characteristics, OI was initially classified as type I (mild), type II (lethal), type III (severe), and type IV (moderate).^6^ However, the International Society of Skeletal Dysplasia suggested adding type V, which is characterized by the formation of calcification in interosseous membranes.^1 , 2 , 5^

Altered bone growth often leads to maxillary hypoplasia, predisposing individuals with this condition to the development of Angle class III malocclusion and anterior crossbite.^7^ Changes in dental development are also frequent in individuals with OI. Structure alteration of the dentin, acknowledged as dentinogenesis imperfecta (DI) is one of the most common features noted in individuals with OI.^1 - 3 , 5 , 6^ Other dental alterations during tooth development may also result in tooth variations in number, form, and position.^8^

It is important for individuals with OI to have access to prevention programs in oral health since they are more vulnerable to dental caries.^9 - 10^ The occurrence of dental alterations can hinder some dental procedures, such as endodontic treatment. Such anomalies can also cause pain, sensitivity, altered speech, and chewing, as well as occlusal and esthetic problems.

Therefore, identifying these problems in individuals with OI can assist dentists in planning treatment while reducing the associated clinical consequences, the need for complex procedures, and the financial cost of treatment.

No systematic reviews have summarized data on the association between dental anomalies and OI yet. Considering the existing body of knowledge on this issue, compilation of data is necessary for the evaluation of the state of knowledge on a specific topic.^11^ The literature already points to a strong relationship between DI and OI;^1 , 2 , 5 , 6^ thus, DI data have not been addressed in this systematic review and meta-analysis. This study focuses on the investigation of other dental alterations that may be associated with OI. In this context, the compilation of such data will provide healthcare professionals with information on possible dental anomalies that could be identified when providing care for patients with OI.

Therefore, this systematic review and meta-analysis aims to describe the prevalence of dental anomalies, except for DI, in individuals with OI. Moreover, the objective was to compare the prevalence of dental anomalies between individuals with and without OI, and the prevalence of dental anomalies between individuals with different types of OI.

## Methodology

### Protocol and registration

The Meta-analyses Of Observational Studies in Epidemiology (MOOSE)^12^ checklist was used to report this systematic review and meta-analysis. The MOOSE is the most recommended checklist for reporting meta-analysis of observational studies in Epidemiology.^13^ A protocol was registered in the International Prospective Register of Systematic Reviews (PROSPERO) under registration number CRD42020213324.

### Eligibility criteria

Inclusion criteria were observational studies (with and without a comparison group) that evaluated the prevalence of dental alterations in individuals with OI. No restrictions were imposed regarding language or year of publication. Studies with insufficient data to calculate the prevalence of dental anomalies and case reports; literature, integrative, and scoping reviews; conference abstracts; book chapters; and protocols were excluded. In this systematic review and meta-analysis, data on DI were not considered. The PECO question was as follows:

P (Population) = individuals (any sex and age).

E (Exposure) = individuals with OI (any type).

C (Comparison) = individuals without OI, individuals with different types of OI, or no comparison group.

O (Outcomes) = prevalence and comparative data on dental anomalies (ultrastructural, macroscopic, and/or radiographic) except for a positive diagnosis of DI.

### Databases and research strategy

Searches were conducted in the following databases: PubMed (National Library of Medicine), Web of Science (Clarivate Analytics), Scopus (Elsevier), and Ovid (Wolters Kluwer). The search strategy used in each database can be seen in Supplementary File 1 . Searches were conducted from databases’ date of inception until October 2022. Grey literature was searched using ProQuest and Google Scholar, with the searches in each database limited to the first 200 references.^14^ In ProQuest and Google Scholar, the first 200 references were sorted by relevance. Finally, further articles were manually searched in the reference lists of the obtained studies.

Duplicate references from different databases were identified and removed using the Mendeley program (Mendeley Desktop Software; V-1.17.10). Then, the references were imported into the Rayyan web software (Qatar Computing Research Institute, Doha, Qatar), an electronic application for systematic reviews, which assists in the selection of abstracts.

### Selection of studies

Two researchers (HVP and ECBS) performed the selection of the articles independently. In Step 1, the titles and abstracts were evaluated for the pre-selection of articles. Those considered potentially eligible were then submitted to full-text analysis by the two researchers in Step 2. References that met the eligibility criteria in Step 2 were included. In cases of disagreement between the two researchers regarding the eligibility criteria of a given article, a third researcher (NCRC) was responsible for deciding.

### Data extraction and extracted items

Two researchers (HVP and ECBS) extracted data from the selected articles. In cases of disagreement between the two researchers, such as the content of the data extracted or typing errors, a discussion was held. A third researcher (NCRC) was responsible for the final decision regarding the data extraction if the divergences persisted. Among the three researchers involved in the selection of the studies and data extraction, two (ECBS and NCRC) are specialists in Orthodontics and the three researchers work in a service where oral healthcare is provided to individuals with special care needs.

Collected data included name of authors, year of publication, country where the study was conducted, place where participants were recruited, sample size, age, sex, type of participants’ OI, dental alterations evaluated, and results of the evaluation of the individuals with OI regarding occurrence of dental alterations. When the study included a control group of individuals without OI, the comparisons between groups were also assessed.

### Quality assessment of included studies

The assessment of the methodological quality of the included studies was conducted with critical evaluation checklists from The Joanna Briggs Institute for prevalence studies^15^ and case-control studies,^16^ independently. In general, the tools used consist of questions about the sample size, participants, place of recruitment, data analysis, use of valid methods to identify the assessed condition, the reliability of the method used to assess the condition, appropriate statistical analysis, and the study’s response rate. Answers to each item included “yes (high quality),” “unclear (uncertain quality),” “no (low quality),” or “not applicable.”

The quality in each study was classified as low if the study scored up to 49% of the items with “yes”; moderate if the study scored from 50% to 69%; and high if the study scored more than 70%.

### Synthesis of results

Meta-analyses of proportion were conducted using the software MedCalc (MedCalc Software bv, Ostend, Belgium; https://www.medcalc.org; 2020). The prevalence of agenesis, bulbous crown, discoloration, ectopic eruption, impaction of second molars, tooth impaction, microdontia, pulp obliteration, and taurodontism among individuals with OI was assessed. Statistical heterogeneity was assessed by means of the *I* ^2^ statistics.^17^ The random effect model was employed. Percentage values and 95% confidence intervals (CI) were provided.

The software Review Manager (Review Manager (RevMan) (Computer program) version 5.4, The Cochrane Collaboration, 2020) was used to compare the prevalence of tooth impaction between individuals with and without OI. Comparisons between individuals with OI type I, with OI type III, and with OI type IV were also conducted. Statistical heterogeneity was assessed by means of the *I* ^2^ statistics.^17^ The random effect model was employed. Odds ratio (OR) and 95% CI were provided. The researcher who conducted the meta-analysis (LGA) is a specialist in Orthodontics.

### Strength of the evidence assessment

The strength of evidence from the selected studies for the meta-analyses was assessed using of the Grading of Recommendations Assessment, Development and Evaluation (GRADE) system.^18^ The summary of results was performed according to GRADEpro (www.gradepro.org).

GRADEpro is used to assess certainty of evidence and to evaluate results from meta-analyses of randomized clinical trials and observational studies. In the assessment of observational studies, evaluation of cohort case-control, cross-sectional with a control group, and case series is feasible. Therefore, GRADEpro was used to improve the adopted method and the quality of evidence.

The components of the GRADE^18^ framework were used to assess the overall quality of the available evidence of dental anomalies in individuals with OI, evaluating risk of bias (overall study limitations of the evidence identified), inconsistency (unexplained heterogeneity or variability in results across studies with differences of results), indirectness (if the study sample and prevalence estimate in the primary studies do not accurately reflect the PECO; is there generalization of the results?), imprecision (few studies and small number of participants across studies), and publication bias.

## Results

### Selection of studies

**Figure 1:**
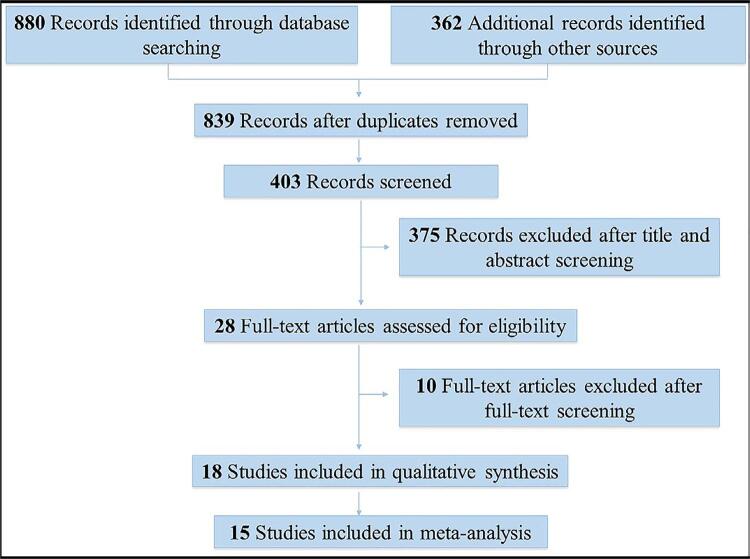
Diagram showing the process of study selection in the systematic review

The searches in the electronic databases retrieved 1,242 references. After exclusion of 403 duplicates, the titles and abstracts of 375 references were analyzed in Step 1. A total of 28 references were considered potentially eligible and submitted to full-text analysis in Step 2. Then, 18 observational studies met the eligibility criteria and were included in this systematic review and meta-analysis.^19 - 36^Supplementary File 2 shows the reason for the exclusions in Step 2. The full text of the study of Lund, et al.^20^ (1998) was unavailable. The corresponding author was contacted, who provided the full version of the study. Figure 1 shows the flowchart of the selection process of the articles.

### Characteristics of studies included

The 18 studies evaluated a total of 1,221 individuals with OI and 185 without OI. The articles were published from 1984 to 2021. All were published in English, except for the study of Alania, et al.^24^ (2011), which was published in Russian. Five studies were conducted in North America: Canada^19 , 32 , 33^ and USA^21^ and Canada/USA.^30^ Nine studies were conducted in Europe: Denmark,^20 , 29^ Sweden,^22 , 26 - 28 , 34^ Georgia,^24^ France/Belgium.^23^ One study was conducted in South America: Brazil.^31^ Two studies were conducted in Africa: Egypt^25^ and South Africa.^36^ One study was conducted in Asia: Vietnam.^35^

Considering the participants involved in the included studies, 15 evaluated only individuals with OI,^19 , 21 , 22 , 24 - 30 , 32 - 36^ whereas three studies evaluated a group of individuals with OI and a control group (individuals without OI).^20 , 23 , 31^

Individuals with OI were recruited from university centers,^20 , 21 , 24 , 31^ specialized treatment centers,^23 , 25 , 28 , 30 , 36^ and hospitals.^19 , 22 , 26 , 27 , 29 , 32 - 35^ The participants from the control groups (without OI) were recruited from university centers^31^ and the French general population.^23^ In the study of Lund, et al.^20^ (1998), the place of recruitment of the comparison group was not reported.

All age groups were included in the studies. Furthermore, dental anomalies of number,^21 , 22 , 27 , 28 , 30 - 32 , 34 , 35^ form,^19 , 20 , 22 , 24 - 26 , 29 - 31 , 33 , 34 , 36^ structure,^19 , 20 , 22 , 25 , 28 - 31 , 33^ and location^19 , 21 , 22 , 25 , 26 , 28 , 30 - 32 , 35^ were assessed. Four studies evaluated the delay in tooth eruption.^21 , 23 , 25 , 35^

The presence of dental alterations was evaluated by means of clinical examination,^19 - 24 , 26 , 28 - 30 , 32 - 34 , 35^ radiographic examination,^19 - 22 , 24 , 26 - 31 , 34 - 36^ and photographs.^20 , 26 , 29 , 32 , 33^ One study did not clearly describe how the assessments of dental alterations were performed.^25^

### Results of the individual studies included

#### Dental anomalies of number

Nine studies evaluated the presence of tooth agenesis in individuals with OI.^21 , 22 , 27 , 28 , 30 - 32 , 34 , 35^ Tooth agenesis in the maxilla ( *p* =0.003) and in the mandible ( *p* =0.046) was more frequent in individuals with OI than without OI.^31^ Individuals with OI type III and IV were significantly more affected by tooth agenesis when compared with OI type I.^32^

#### Dental anomalies of form

A total of three studies evaluated the presence of microdontia in individuals with OI.^25 , 31 , 34^ Microdontia was more frequent in individuals with OI when compared to without OI ( *p* =0.001).^31^

Furthermore, seven studies evaluated the prevalence of taurodontism.^22 , 26 , 29 - 31 , 33 , 36^ This dental anomaly was more frequent in OI individuals when compared to without OI ( *p* =0.034).^31^ Two studies found no difference between individuals with OI type I and OI type III/IV regarding the prevalence of taurodontism.^29 , 33^

The shape of the roots (short thin and curved) was evaluated in five studies.^20 , 24 , 29 - 31^ Individuals with OI had a higher prevalence of narrow roots when compared to individuals without OI of the same sex and age ( *p* <0.001).^31^ The presence of short roots was more common in individuals with OI type III/IV (severe-moderate) than in individuals with OI type I (mild) ( *p* <0.001).^29^

The prevalence of cervical constriction and bulbous crown was evaluated in six studies.^19 , 24 , 29 - 31^ It was more prevalent in individuals with OI ( *p* <0.001),^31^ especially in individuals with OI type III/IV (moderate-severe) ( *p* <0.001).^29^

Other types of dental anomalies of form were also evaluated in the studies included in this systematic review and meta-analysis, such as root dilaceration,^31^ *dens invaginatus* ,^22 , 34^ and *dens evaginatus* .^34^

#### Dental anomalies of structure

Anomalies were reported in the three dental structures: pulp, dentin, and enamel. Eight studies evaluated the prevalence of pulp obliteration.^19 , 20 , 22 , 28 - 31 , 33 , 35^ Pulp obliteration was more prevalent in individuals with OI type III/IV (moderate-severe) than in individuals with OI type I (mild).^29 , 33^ Three studies investigated the presence of calcification (denticles) inside the pulp chamber or root canals.^20 , 22 , 29^

Tooth discoloration was investigated in five studies.^19 , 20 , 29 , 30 , 33^ Regarding tooth enamel, two studies evaluated the presence of developmental defects of enamel (hypocalcification and hypomineralization).^25 , 34^

#### Dental anomalies of location

Ectopic eruption was investigated in three studies.^21 , 22 , 31^ Individuals with OI had a higher prevalence of ectopic tooth when compared to individuals without OI of the same sex and age ( *p* <0.049).^31^ The presence of dental transposition was found.^22^ Dental impaction was investigated in eight studies.^19 , 21 , 22 , 27 , 30 - 32 , 35^

Supplementary File 3 presents the characteristics and results of the included studies.

## Quality assessment in individual studies

Furthermore, eight (47.0%) studies were classified as low quality,^19 - 22 , 24 , 25 , 29 , 30^ two studies (6%) as moderate quality,^21 , 27^ and eight (47.0%) as high quality.^25 , 28 , 31 - 36^ The following characteristics contributed to the low quality: lack of validated, referenced instruments for the diagnosis of the evaluated characteristics, lack of calibration of the examiners, and lack of a sample size calculation. Supplementary Files 4 and 5 present further details on the quality assessment of the included studies.

## Synthesis of results

**Figure 2:**
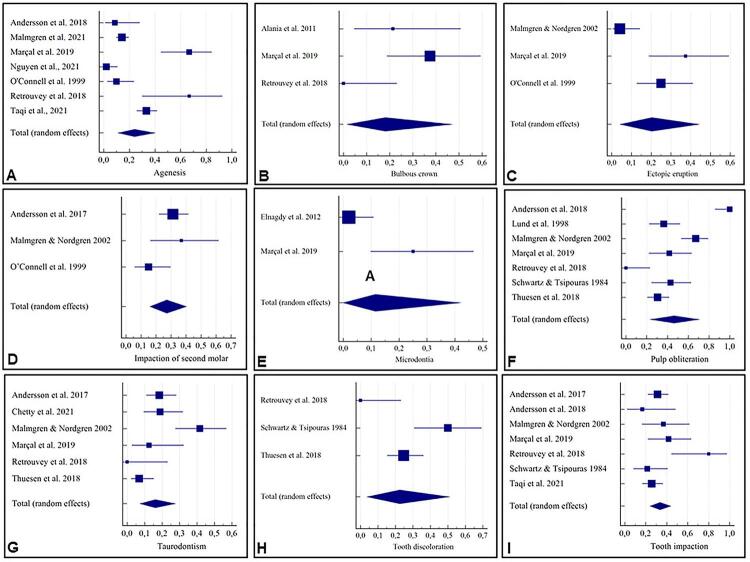
Forest plot of meta-analysis for the studies evaluating prevalence of dental anomalies in individuals: (A) Agenesis; (B) Bulbous crown; (C) Ectopic eruption; (D) Impaction of second molar; (E) Microdontia; (F) Pulp obliteration; (G) Taurodontism; (H) Tooth discoloration; (I) Tooth impaction

Figure 2 provides the results of the meta-analyses of prevalence of agenesis (23.99%), bulbous crown (18.11%), ectopic eruption (20.39%), impaction of second molars (27.12%), microdontia (11.52%), pulp obliteration (46.43%), taurodontism (16.10%), discoloration (22.42%), and tooth impaction (33.53%) among individuals with OI. The random effect model was used.

**Figure 3:**
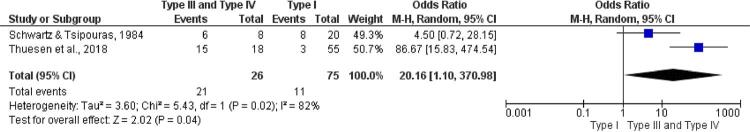
Forest plot of meta-analysis for the studies evaluating dental impaction in individuals with and without OI

**Figure 4:**
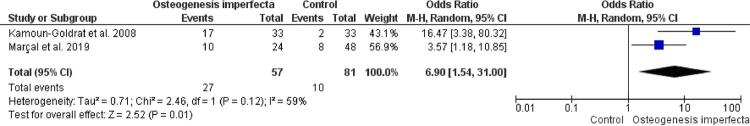
Forest plot of meta-analysis for the studies evaluating discoloration in individuals with OI type III and type IV versus type I

**Figure 5:**
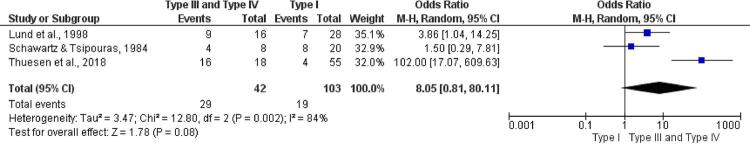
Forest plot of meta-analysis for the studies evaluating pulp obliteration in individuals with OI type III and type IV versus type I

Individuals with OI were 6.90 times more likely to present tooth impaction than individuals without (CI=1.54 – 31.00, *I* ^2^=59%) ( Figure 3 ). Individuals with OI type III and IV were 20.16 times more likely to present tooth discoloration than individuals with OI type I (CI=1.10 – 370.98, *I* ^2^=82%) ( Figure 4 ). For pulp obliteration, the results were not statistically significant, but the prevalence of this condition among individuals with OI type III and IV was higher than among individuals with OI type I (CI=0.81 – 80.11, *I* ^2^=84%) ( Figure 5 ). The random effect model was used.

## Strength of the evidence assessment

In the meta-analyses of prevalence, based on the GRADE evaluation, the strength of evidence was classified as “ *Low* ” for evaluation of four dental anomalies (agenesis, impaction of second molars, tooth impaction, and taurodontism) and “ *Very low* ” for another five anomalies (bulbous crown, discoloration, ectopic eruption, microdontia, and pulp obliteration). The meta-analysis that compared the prevalence of tooth impaction between individuals with and without OI was classified as *“Very Low,”* as well as the meta-analyses that compared discoloration and pulp obliteration between individuals with OI type III/IV versus OI type I ( Supplementary File 6 ).

## Discussion

Individuals with OI have a greater likelihood of dental alterations compared to typical individuals. Dental problems can affect quality of life, chewing function, and social life, as well as cause pain, increased sensitivity, and esthetic problems.

In this systematic review and meta-analysis, data on the prevalence of a positive diagnosis of DI in individuals with OI were not included. Although not all individuals with OI exhibit DI, there is a strong association between both conditions that has already been well discussed in the literature. Shields, Bixler, and el-Kafrawy^37^ (1973) proposed a classification of DI with three types, being type 1 associated with OI. Thus, it is important to investigate other dental alterations that might be associated with OI, informing oral health practitioners and any other medical professionals who provide healthcare services to this population.

Despite the low strength of evidence, this systematic review and meta-analysis found that individuals with OI have a greater likelihood of tooth impaction in comparison to individuals without OI. This probably occurs, in part, due to the lack of space caused by growth problems of the maxilla, which is very common in OI,^7^ leading to a greater occurrence of impacted maxillary second molars.^26^ Another factor that may contribute to tooth impaction is the excessive bulbosity or volume of the crowns, which impairs the eruption process.^21^ Genetic analysis studies indicate that impacted teeth in individuals with OI are also associated with qualitative mutations in the *COL1A1* / *COL1A2* genes.^26 , 32^

Drug therapy with bisphosphonates is part of the treatment of children with OI. Bisphosphonates considerably improve the quality of life of affected individuals by reducing bone pain and increasing bone mineral density.^38 , 39^ However, treatment seems to present no positive effect on dental alterations in this population^23 , 34^ since part of the dose of intravenously administered bisphosphonates is deposited in the skeleton at sites of active bone remodeling.^40^ Tooth development and eruption are accompanied by remodeling of adjacent bone, with deposition by osteoblasts and reabsorption by osteoclasts. Moreover, serum calcium and phosphorus are attracted to form hydroxyapatite in the process of the development of tooth structure.^41^ Thus, bisphosphonates can affect tooth eruption and the occurrence of impacted teeth, microdontia, and tooth agenesis.^23 , 31 , 34 , 42^

Dentin is produced by odontoblasts as predentin, a mesenchymal product composed of collagen fibers and phosphoprotein.^43^ Due to mutations in the genes that encode type I collagen, defects in the dentin can occur in individuals with OI, such as the non-formation of the tooth.^22 , 27^ The composition of bone and dentin is similar, but some fundamental physiological differences exist.^26^ Dentin presents no osteoclasts or continual remodeling. Therefore, the effects of bisphosphonates on bone tissue are not applicable to dentin.

Individuals with severe and moderate OI (types III and IV) are more likely to develop discoloration compared with mild OI (type I), indicating an association between this dental abnormality and the severity of the OI phenotype. Studies have shown that this condition occurs due to the qualitative mutation in type I collagen. Independently of the severity of OI, discoloration mainly affects teeth with a thinner enamel (anterior and mandibular teeth).^20 , 33^

According to Taqi, et al.^33^ (2021), tooth discoloration is associated with pulp canal obliteration in individuals with OI, and the risk of developing both conditions increases when teeth are out of contact. Also, evidence suggests that tooth discoloration is more related to enamel thickness and a thinner enamel may increase the translucency of the discolored dentin.^33^ The lack of occlusal forces may stimulate odontoblasts to secrete more dentin in a progressive, immature way, resulting in pulp canal obliteration and tooth discoloration. Thus, the hypothesis is raised that the oral environment plays a role in structural dental anomalies, which may indicate that odontoblasts present mechanical receptors that respond to mechanical stress.

No correlation has been found between taurodontism and any type of mutation in the *COL1A1* / *COL1A2* genes in this population.^26 , 33^ Thus, the cause of this condition may not be related to a specific type of collagen abnormality.^26^ Children with OI generally exhibit a more severe form of taurodontism compared with children diagnosed with some type of syndrome.^26^ One study suggests that taurodontism in individuals with OI is likely associated with delayed pulp maturation.^33^ Odontoblastic deficiency and alterations in Hertwig’s epithelial root sheath can also lead to the formation of taurodontic roots.^36 , 44^ The occurrence of taurodontism is asymptomatic and can pose a challenge in cases that require endodontic treatment.^44^

The early diagnosis of dental anomalies is important to guide the development of the occlusion, and failure to do so could lead to greater harm. The findings of this systematic review and meta-analysis can be useful to dentists during clinical appointments for informing parents about the oral health status of their children. The information may also be helpful for the dentist in recognizing individuals with a possible diagnosis of OI, allowing earlier referrals when necessary. The clinical and radiographic evaluation of children and adolescents with OI should be discerning and continual throughout life to determine the correct diagnosis and allow the early identification of potential dental and occlusal complications.

The studies included in this systematic review and meta-analysis presented some methodological issues. The main limitation was the lack of representativeness in the cases. The calculation of the sample size was not described in any of the studies. Methodological differences regarding the type of dental anomaly, the absence of a control group, and stratification of the sample by the type of OI impeded the incorporation of all articles in our study. Further studies with robust methods, representative samples, and measures to control for confounding factors are needed. Methodological adjustments are necessary to investigate whether the association between dental anomalies and OI may be influenced by other factors.

## Conclusion

Pulp obliteration, dental impaction, and tooth agenesis were the most prevalent dental anomalies in the OI group. Individuals with OI were more likely to have dental impaction than individuals without OI. Individuals with OI type III (severe) and IV (moderate) are more likely to have tooth discoloration than individuals with OI type I (mild).

## References

[1] Van Dijk FS, Sillence DO (2014). Osteogenesis imperfecta: clinical diagnosis, nomenclature and severity assessment. Am J Med Genet A.

[2] Valadares ER, Carneiro TB, Santos PM, Oliveira AC, Zabel B (2014). What is new in genetics and osteogenesis imperfecta classification?. J Pediatr.

[3] Hoyer-Kuhn H, Netzer C, Semler O (2015). Osteogenesis imperfecta: pathophysiology and treatment. Wien Med Wochenschr.

[4] Holmes DF, Lu Y, Starborg T, Kadler KE (2018). Curr Top Dev Biol.

[5] Subramanian S, Anastasopoulou C, Viswanathan VK (2023). StatPearls.

[6] Sillence D, Senn A, Danks D (1979). Genetic heterogeneity in osteogenesis imperfecta. J Med Genet.

[7] Prado HV, Teixeira SA, Rabello F, Vargas-Ferreira F, Borges-Oliveira AC, Abreu LG (2022). Malocclusion in individuals with osteogenesis imperfecta: a systematic review and meta-analysis. Oral Dis.

[8] Salerno C, D'Avola V, Oberti L, Almonte E, Bazzini EM, Tartaglia GM (2021). Rare genetic syndromes and oral anomalies: a review of the literature and case series with a new classification proposal. Children (Basel).

[9] Prado HV, Carneiro NC, Perazzo MF, Abreu MH, Martins CC, Borges-Oliveira AC (2019). Assessing a possible vulnerability to dental caries in individuals with rare genetic diseases that affect the skeletal development. Orphanet J Rare Dis.

[10] Debossan SA, Deps TD, Prado HV, Abreu MH, Borges-Oliveira AC (2022). Access to oral health care services for individuals with rare genetic diseases affecting skeletal development. Spec Care Dentist.

[11] Meerpohl J, Herrle F, Reinders S, Antes G, von Elm E (2012). Scientific value of systematic reviews: survey of editors of core clinical journals. PLoS One.

[12] Stroup DF, Berlin JA, Morton SC, Olkin I, Williamson GD, Rennie D (2000). Meta-analysis of observational studies in epidemiology: a proposal for reporting. Meta-Analysis of Observational Studies in Epidemiology (MOOSE) group. JAMA.

[13] van Zuuren EJ, Fedorowicz Z (2016). MOOSE on the LOOSE: checklist for meta-analyses of observational studies. Br J Dermatol.

[14] Haddaway NR, Collins AM, Coughlin D, Kirk S (2015). The role of google scholar in evidence reviews and its applicability to grey literature searching. PLoS One.

[15] Munn Z, Moola S, Lisy K, Riitano D, Tufanaru C (2015). Methodological guidance for systematic reviews of observational epidemiological studies reporting prevalence and incidence data. Int J Evid Based Healthc.

[16] Moola S, Munn Z, Tufanaru C, Aromataris E, Sears K, Sfetcu R, Aromataris E, Munn Z (2020). JBI Manual for Evidence Synthesis.

[17] Deeks JJ, Higgins JP, Altman DG, Higgins JP, Thomas J, Chandler J, Cumpston M, Li T, Page MJ (2022). Cochrane Handbook for Systematic Reviews of Interventions version 6.3 (updated February 2022).

[18] Guyatt GH, Oxman AD, Vist GE, Kunz R, Falck-Ytter Y, Alonso-Coello P (2008). GRADE: an emerging consensus on rating quality of evidence and strength of recommendations. BMJ.

[19] Schwartz S, Tsipouras P (1984). Oral findings in osteogenesis imperfecta. Oral Surg Oral Med Oral Pathol.

[20] Lund A, Jensen B, Nielsen L, Skovby F (1998). Dental manifestations of osteogenesis imperfecta and abnormalities of collagen I metabolism. J Craniofac Genet Dev Biol.

[21] O’Connell A, Marini J (1999). Evaluation of oral problems in an osteogenesis imperfecta population. Oral Surg Oral Med Oral Pathol Oral Radiol Endod.

[22] Malmgren B, Nordgren S (2002). Dental aberrations in children and adolescents with osteogenesis imperfecta. Acta Odontol Scand.

[23] Kamoun-Goldrat A, Ginisty D, Le Merrer M (2008). Effects of bisphosphonates on tooth eruption in children with osteogenesis imperfecta. Eur J Oral Sci.

[24] Alania KN, Iverieli MB, Abashidze NO, KhB Gogishvili, Chigladze TT (2011). Oral cavity features in patients suffering from osteogenesis imperfect. Georgian Med News.

[25] Elnagdy G, ELRefaiey M, Aglan M, Ibrahim R, ELBadry T (2012). Oro-dental manifestations in different types of osteogenesis imperfect. Austr J Basic Appl Sci.

[26] Andersson K, Dahllöf G, Lindahl K, Kindmark A, Grigelioniene G, Åström E (2017). Mutations in COL1A1 and COL1A2 and dental aberrations in children and adolescents with osteogenesis imperfecta - A retrospective cohort study. PLoS One.

[27] Malmgren B, Andersson K, Lindahl K, Kindmark A, Grigelioniene G, Zachariadis V (2017). Tooth agenesis in osteogenesis imperfecta related to mutations in the collagen type I genes. Oral Dis.

[28] Andersson K, Malmgren B, Åström E, Dahllöf G (2018). Dentinogenesis imperfecta type II in Swedish children and adolescents. Orphanet J Rare Dis.

[29] Thuesen K, Gjørup H, Hald J, Schmidt M, Harsløf T, Langdahl B (2018). The dental perspective on osteogenesis imperfecta in a Danish adult population. BMC Oral Health.

[30] Retrouvey J, Taqi D, Tamimi F, Dagdeviren D, Glorieux F, Lee B (2019). Oro-dental and cranio-facial characteristics of osteogenesis imperfecta type V. Eur J Med Genet.

[31] Marçal FF, Ribeiro EM, Costa FW, Fonteles CS, Teles GS, Barros Silva PG (2019). Dental alterations on panoramic radiographs of patients with osteogenesis imperfecta in relation to clinical diagnosis, severity, and bisphosphonate regimen aspects: a STROBE-compliant case-control study. Oral Surg Oral Med Oral Pathol Oral Radiol.

[32] Taqi D, Moussa H, Schwinghamer T, Vieira AR, Dagdeviren D, Retrouvey JM (2021). Missing and unerupted teeth in osteogenesis imperfecta. Bone.

[33] Taqi D, Moussa H, Schwinghamer T, Ducret M, Dagdeviren D, Retrouvey JM (2021). Osteogenesis imperfecta tooth level phenotype analysis: cross-sectional study. Bone.

[34] Malmgren B, Thesleff I, Dahllöf G, Åström E, Tsilingaridis G (2021). Abnormalities in tooth formation after early bisphosphonate treatment in children with osteogenesis imperfecta. Calcif Tissue Int.

[35] Nguyen HT, Vu DC, Nguyen DM, Dang QD, Tran VK, Le H (2021). Dentinogenesis imperfecta and caries in osteogenesis imperfecta among Vietnamese children. Dent J (Basel).

[36] Chetty M, Roomaney IA, Beighton P (2021). Taurodontism in dental genetics. BDJ Open.

[37] Shields ED, Bixler D, l-Kafrawy AM (1973). A proposed classification for heritable human dentin defects with a description of a new entry. Arch Oral Boil.

[38] Yazan H, Güneş N, Akpınar E, Özyalvaç ON, Uludağ Akkaya D, Tuysuz B (2021). Effects of long-term pamidronate treatment on bone density and fracture rate in 65 osteogenesis imperfecta patients. Turk Arch Pediatr.

[39] Dlesk TE, Larimer K (2022). Multimodal pain management of children diagnosed with osteogenesis imperfecta: an integrative literature review. Pain Manag Nurs.

[40] Cremers S, Drake MT, Ebetino FH, Bilezikian JP, Russell RG (2019). Pharmacology of bisphosphonates. Br J Clin Pharmacol.

[41] Wise GE, King GJ (2008). Mechanisms of tooth eruption and orthodontic tooth movement. J Dent Res.

[42] Malmgren B, Tsilingaridis G, Monsef-Johansson N, Qahtani ZHA, Dahllöf G, Åström E (2020). Bisphosphonate therapy and tooth development in children and adolescents with osteogenesis imperfecta. Calcif Tissue Int.

[43] Farci F, Soni A (2022). StatPearls.

[44] Dineshshankar J, Sivakumar M, Balasubramanium AM, Kesavan G, Karthikeyan M, Prasad VS (2014). Taurodontism. J Pharm Bioallied Sci.

